# Nanostructured Cellulose-Based Aerogels: Influence of Chemical/Mechanical Cascade Processes on Quality Index for Benchmarking Dye Pollutant Adsorbents in Wastewater Treatment

**DOI:** 10.3390/gels9120958

**Published:** 2023-12-06

**Authors:** Annachiara Pirozzi, Esther Rincón, Eduardo Espinosa, Francesco Donsì, Luis Serrano

**Affiliations:** 1 Department of Industrial Engineering, University of Salerno, Via Giovanni Paolo II, 132, 84084 Fisciano, Italy; fdonsi@unisa.it; 2 BioPrEn Group (RNM 940), Chemical Engineering Department, Instituto Químico para la Energía y el Medioambiente (IQUEMA), Faculty of Science, Universidad de Córdoba, 14014 Córdoba, Spain; esther.rincon@uco.es (E.R.); eduardo.espinosa@uco.es (E.E.)

**Keywords:** agri-food residues, nanocellulose, TEMPO oxidation, high-pressure homogenization, wastewater treatment, methylene blue, dyes

## Abstract

(1) Background: Nanostructured cellulose has emerged as an efficient bio-adsorbent aerogel material, offering biocompatibility and renewable sourcing advantages. This study focuses on isolating (ligno)cellulose nanofibers ((L)CNFs) from barley straw and producing aerogels to develop sustainable and highly efficient decontamination systems. (2) Methods: (Ligno)cellulose pulp has been isolated from barley straw through a pulping process, and was subsequently deconstructed into nanofibers employing various pre-treatment methods (TEMPO-mediated oxidation process or PFI beater mechanical treatment) followed by the high-pressure homogenization (HPH) process. (3) Results: The aerogels made by (L)CNFs, with a higher crystallinity degree, larger aspect ratio, lower shrinkage rate, and higher Young’s modulus than cellulose aerogels, successfully adsorb and remove organic dye pollutants from wastewater. (L)CNF-based aerogels, with a quality index (determined using four characterization parameters) above 70%, exhibited outstanding contaminant removal capacity over 80%. The high specific surface area of nanocellulose isolated using the TEMPO oxidation process significantly enhanced the affinity and interactions between hydroxyl and carboxyl groups of nanofibers and cationic groups of contaminants. The efficacy in adsorbing cationic dyes in wastewater onto the aerogels was verified by the Langmuir adsorption isotherm model. (4) Conclusions: This study offers insights into designing and applying advanced (L)CNF-based aerogels as efficient wastewater decontamination and environmental remediation platforms.

## 1. Introduction

In 2019, global food waste, also referred to as agri-food residues (AFRs), reached approximately 931 million tons along the entire supply chain, originating primarily from household consumption (61%), food service (26%), and retail (13%), leading to significant negative impacts on the environment, economy, and society. In response to this issue, UN’s 2030 Agenda [1], specifically Sustainable Development Goal 12.3, emphasized the need to halve food waste and reduce food loss by 2030. As awareness of our impact on the planet grows, the food and agricultural industries face challenges related to excessive natural resource consumption, soil degradation, environmental pollution, and depletion of food resources. Consequentially, new policies and efforts toward the design of appropriate management of AFRs have to be put in place to contribute to a sustainable development, respectful of the social and environmental facets [2]. Within this frame, the exploitation of AFRs presents new opportunities to extract and purify high value-added compounds [3,4,5], including fibers, antioxidant phytochemicals, oligosaccharides, vitamins, pectin, enzymes, pigments, and organic acids inter alia, of particular interest to innovative applications in different fields (i.e., food ingredients, nutraceuticals, cosmeceutical, bioderived fine chemicals, biofuels, etc.) [6]. By recovering compounds from AFRs, residues can be reduced, and economic profitability can be increased [7]. Therefore, the partial (or even total) usage of industrial and agricultural waste could be beneficial both environmentally and economically by reducing their environmental burden [8], and at the same time solving the economic issues [9].

Among side streams, lignocellulosic biomass derived from forestry, agriculture, agro-industrial activities, and food waste is the most abundant, renewable, and cost-effective biomass on Earth, with an annual production of around 200 billion tons [10,11,12,13,14]. Crop residues, particularly straws from cereal plants like barley, oat, rye, and wheat, contribute 74% of this biomass [15]. These biomasses primarily consist of cellulose (34–40%), hemicellulose (20–25%), lignin (20%), and other minor components [16]. Cellulose, a renewable organic carbohydrate biopolymer with a unique molecular structure, possesses notable chemical (hydrophilicity, chirality, degradability, and chemical variability) and mechanical (tensile strength and Young’s modulus) properties [17]. Such promising properties have attracted increasing interest as constitutive elements for developing biomaterials, including nanofillers or additives [18], adsorption materials [19], and stabilizers of Pickering emulsions [20], especially through advanced nanotechnology tools [21]. Owing to its hierarchical organization in a supramolecular structure and the hydrogen bonds between hydroxyl groups, as well as its semicrystalline nature, cellulose can be efficiently deconstructed to obtain cellulose nanoparticles (nanocellulose, NC) [22]. NC exhibits desirable physicochemical characteristics, such as high specific surface area and aspect ratio, high crystallinity, purity, excellent mechanical properties, and low thermal expansion and density [17,23,24,25,26]. These features create new and innovative prospects for NC applications in biomedical, environmental, and energy fields [27]. However, due to the wide range of NC applications and its abundance, it is crucial to establish effective and simple comparison methods that assess the suitability of different types of NC for various applications using a single criterion. In this regard, Desmaisons et al. [28] proposed a new method based on determining the NC quality index through multifactorial analysis. This method enables the assessment of the most critical properties of NC for different fields, facilitates NC production monitoring, links energy consumption, and allows comparative evaluation of various suspensions to guide consumers toward the most suitable suspension for a given application.

One of the innovative applications of NC is in the fabrication of ultralight and highly porous aerogels [29,30]. NC-based aerogels exhibit remarkable properties, including high compressive strength (5.2 kPa–16.67 MPa), low specific surface areas (0.05–0.22 g/cm^3^), and ultra-high porosity (93–99%) [31]. Notably, these characteristics are comparable to or even surpass those of synthetic polymer aerogels [32], activated carbon [33], and diatomite-silica aerogels [34], whose application is limited by their poor mechanical [35] and dust-release properties [36]. In contrast to traditional and commercially aerogels, for which materials with near-identical structure and properties are produced with different precursors and drying techniques [37], NC-based aerogels present several advantages: (i) inexhaustible and renewable source, (ii) natural biopolymer with better biodegradability, (iii) the aerogel-making process requires no crosslinking agent due to the abundance of hydroxyl groups, (iv) a stable 3D network structure can be achieved through intramolecular and intermolecular physical crosslinking, and (v) easy chemical modification allows for improvements in mechanical strength and structural characteristics [38]. NC-based aerogel is being applied, among many, in the field of environmental remediation for dye removal from wastewater due to their natural renewability, abundance, ultra-low density, large surface area, and possibility of surface modification [39,40].

Chemicals and dyes are major sources of the environmental contamination of effluent wastewater from various industries [41]. Synthetic dyes, in particular, raise concerns due to their potential toxicity and negative effects on human life and ecosystems. Approximately 10% to 25% of dyes produced annually are discharged into surface water streams during manufacturing and processing operations, with textile industry accounting for nearly 2% of these dyes [42]. In particular, methylene blue, a cationic dye commonly used in textile manufacturing processes for dying cotton, wood, and silk, is a toxic and non-degradable dye [43] that remains stable at elevated temperature, under light exposure, heat, and in the presence of other chemical reactions [44]. Improper wastewater treatment and discharge without adequate management can lead to pollution and various health issues and wellness problems (e.g., eye burns, breathing difficulties, nausea, vomiting, and methemoglobinemia) [45,46,47,48]. Detoxifying toxic dyes from wastewater before discharge is therefore crucial and a very important aspect. Over the recent decades, several conventional techniques for the decolorization of water containing residual dyes have been reported, including physical methods like membrane filtration (nano-filtration, reverse osmosis, electrodialysis) [49], coagulation/flocculation [50], and irradiation [51], as well as chemical and biological methods like advanced oxidation processes [52], electrochemical degradation or ozonation [53], and decolorization by aerobic or anaerobic degradation [54]. Amongst these techniques, adsorption is widely employed due to its (i) effectiveness; (ii) versatility in removing different types of dyes [55,56]; (iii) eco-friendliness; and (iv) absence of harmful substances [57] or secondary contaminants [58]. Natural and synthetic adsorbents, including carbon-based materials, metal oxides, bio-adsorbents, and polymer-based materials, are commonly used for dye removal [59].

This study aims at exploring the advantages of NC-based aerogels as biomaterials with high affinity for specific dyes molecules from wastewater. Specifically, this study establishes the relationship between the quality index of (L)CNFs isolated from barley straw and their effectiveness as aerogels for wastewater decontamination. To achieve this, (L)CNFs were produced through various chemical and mechanical pre-treatments, and their quality index was carefully evaluated. Subsequently, their unique properties enable an efficient detection and adsorption of dye pollutants, with a special emphasis on the kinetic and isothermal analysis of the adsorption process. The novel insights gained from this research contribute significantly to the field of science and technology of NC-based aerogels for environmentally friendly and efficient dyes removal in sustainable solutions for wastewater treatment applications.

## 2. Results and Discussion

### 2.1. Barley Straw Cellulose Fibers Characterization

The chemical constituents of BS, BS-UB, and BS-B are presented in Table 1. Consistent with lignocellulosic materials, BS primarily consists of non-starch polysaccharides, with approximately 35% cellulose, 23% hemicellulose, and 12% lignin. The relatively low lignin content (less than 20%) facilitates its isolation from the fibers and enhances the specific volume, dimensional stability, and rigidity of the pulp [60,61]. After the pulping process, the BS-UB shows a 36% increase in the cellulosic fraction and a slight decrease in hemicellulose, while the lignin content was constant at around 22%. To investigate the impact of residual lignin content on fibrillation behavior [62], a bleaching treatment was carried out. The employed bleaching process effectively and selectively extracted lignin with a yield of 91% without dissolving the hemicellulose, which acts as a physical barrier preventing microfibril aggregation during the homogenization process [63]. The accessory non-structural components of BS include hydrophilic and lipophilic extractives, which are extracted with hot water or organic solvents (such as ethanol), respectively [64], as well as ashes. The pulping process led to a reduction in extractables and ash content. Notably, BS-B exhibited an ash content lower than 1%, making it suitable as a supplement material in pulp production [65].

In addition to yield, the intrinsic viscosity of BS-B, related to the polymer molecular weight according to the Mark–Houwink–Sakurada equation [66], slightly increased after bleaching from 508.15 ± 16.26 mL/g to 646.24 ± 11.21 mL/g and from 1209.87 ± 38.70 mL/g to 1538.66 ± 26.70 mL/g, respectively. The higher degree of polymerization (i.e., the number of glucose monomers forming the cellulose chain) of BS-B compared to BS-UB can be attributed to the degradation effect during the bleaching process.

Along with the remarkable extraction yield and efficient isolation of cellulose, the pulping process enables the production of side streams abundant in valuable compounds. Pirozzi et al. [5] demonstrated that the liquors, after the chemical hydrolysis of tomato pomace biomass, were rich in phenols with antioxidant activity, as well as hemicellulose and lignin particles. Therefore, future studies should address the exploitation and characterization, using chromatographic methods, of the pulping process side streams to recover not only the cellulose pulp but also the valuable compounds present in barley straw AFRs. This will further enhance the overall value proposition of this biorefinery approach.

### 2.2. Quality Index of (L)CNFs and Chemical Characterization

**Figure 1:**
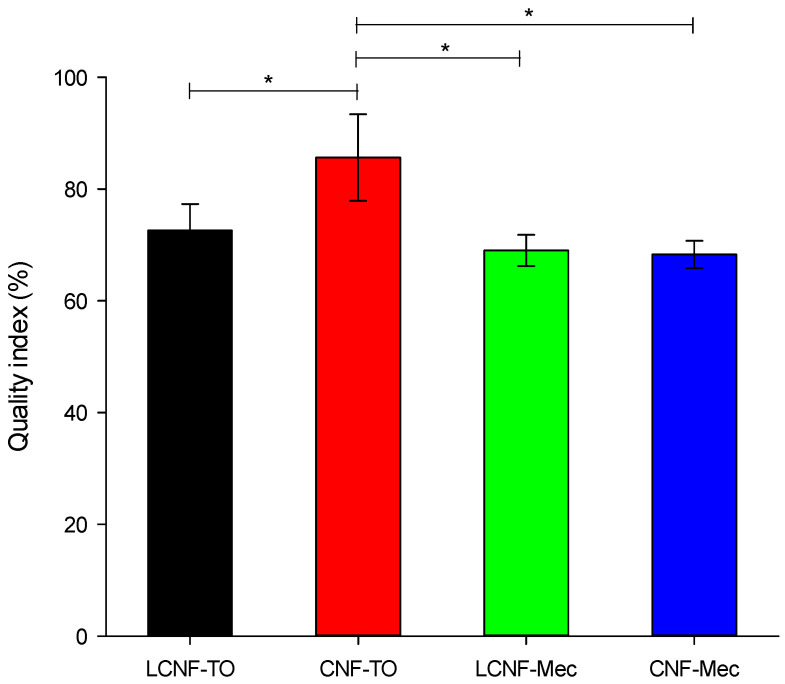
Influence of pre-treatments on (L)CNFs’ quality index. Asterisks denote statistically significant differences (*p* < 0.05).

The quality assessment of nanofibers (evaluated using Equation (5) reported in Section 4.4 of Material and Methods) aimed to (i) establish the relationship between the characteristics of the final (L)CNFs and the employed isolation pre-treatment and (ii) emphasize the impact of their features on the efficacy of cationic contaminant detection and removal. CNF-TO presents a significantly higher QI than those produced from BS-UB with mechanical pre-treatment (Figure 1). The utilization of TEMPO oxidation pre-treatment allowed to produce better quality CNFs due to the increased amount of carboxylic groups, which causes fibers to repeal and repulse [67], thus easing and enabling a more effective performance of the mechanical defibrillation process. Additionally, within the TEMPO-mediated oxidation nanofibers, the one isolated from BS-B demonstrated an elevated level of nanofibrillation, consequently yielding a higher grade of NC quality.

**Figure 2:**
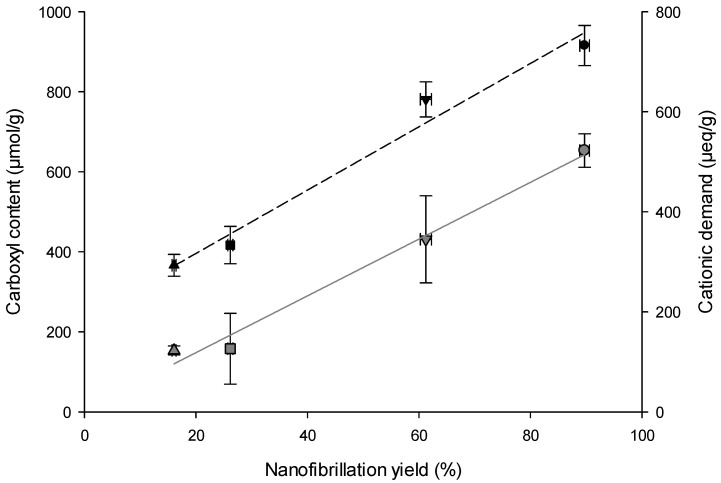
Nanofibrillation yield as a function of the carboxyl content and cationic demand for (△) LCNF-TO, (◻) CNF-TO, (▽) LCNF-Mec, and (○) CNF-Mec. Gray solid line represents the fitting curve of carboxyl content; black dash line represents the fitting curve of cationic demand.

A clear proportional correlation was found between QI and nanofibers properties (Table 2). Notably, CNF-TO exhibits a significantly higher nanofibrillation yield, which can be attributed to the influential role of the applied pre-treatment on the quality of NC isolation. Although the presence of lignin in the cellulose pulp affected the nanofibrillation process, the TEMPO-mediated oxidation treatment resulted in a higher nanofibrillation yield compared to the mechanical pre-treatment. The highest nanofibrillation yield achieved for (L)CNFs-TO is due to the conversion of C6 primary hydroxyl groups into carboxylic groups [68,69], which promoted fiber repulsion and facilitated fibrillation through shearing forces [70]. Both BS-UB and BS-B were subjected to a TEMPO-mediated oxidation pre-treatment and showed a higher carboxyl content compared to the mechanically pre-treated fibers. The increased carboxyl content (280 and 500 μmol/g for LCNF-TO and CNF-TO, respectively) facilitated the fibrillation of cellulose by generating repulsive forces among cellulosic surfaces [71], simultaneously weaking the hydrogen bonding between the microfibrils and increasing fiber hydration and swelling [72]. However, the protonation of carboxyl groups (–COO^−^ into –COOH) led to a reduced surface charge and electrostatic repulsion between nanofibers, resulting in the aggregation of fibrils. The relationship between carboxyl content and nanofibrillation yield is reflected in the colloidal stability of the NCs suspension. A linear curve (f(x) = y_0_ + a·x) adequately describes this relationship, as shown in Figure 2 (and fitting parameters in Table 3). Zeta potential measurements, which reflect the electrokinetic properties of particles in dispersion, indicated that both LCNFs had similar zeta potential values, which were significantly lower (*p* < 0.05) than those of the CNFs (Table 2). Considering this, the zeta potential was mainly influenced by the pulp composition and the pre-treatment applied to isolate the NC. As a consequence, size and shape were found to be the dominant factors in determining the movement of NC in an electric field, with pH having a negligible effect [73]. These results suggest that the negatively charged surface of (L)CNFs could effectively remove a positively charged dye at the basic pH, leading to a higher ionization rate and protonation of the adsorbent’s oxygen and hydroxyl groups. Furthermore, the cationic demand also strongly depends on the nanofibrillation yield, as shown in Figure 2 and Table 3. The nanofibrillation process was more efficient when it resulted in a larger exposed surface area of the cellulosic fibers, which in turn leads to a higher surface charge. The determination of the cationic demand has been evaluated through cationization, which assessed the surface adsorption mechanisms between the –CH_2_–O– groups of cellulose fibers in an alkaline medium and the quaternary ammonium groups of polyDADMAC [74]. By defining the specific surface area of a single polyDADMAC molecule, it would then be possible to theoretically determine the specific surface area and the diameter of NC. CNF-TO showed the lower viscosity value due to the high carboxyl content and yield of fibrillation; therefore, the electrostatic repulsion between the nanofibers reduced [72]. This effect accounts equally for the lowest turbidity and therefore the higher transparency of the CNF-TO.

**Figure 3:**
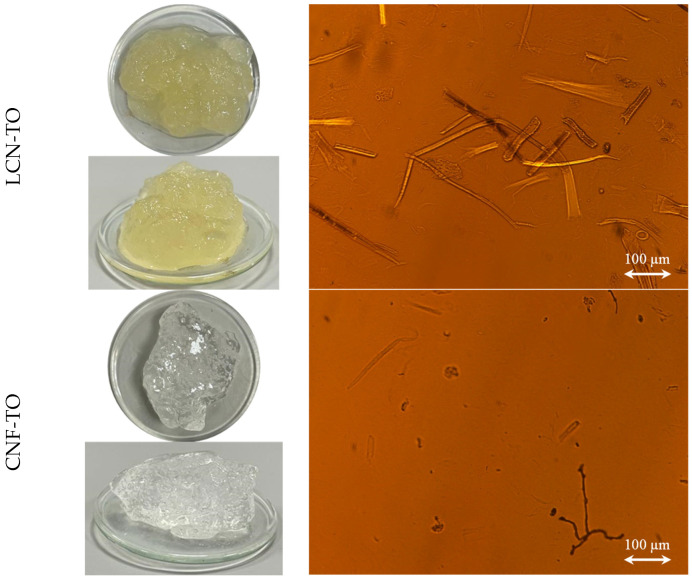

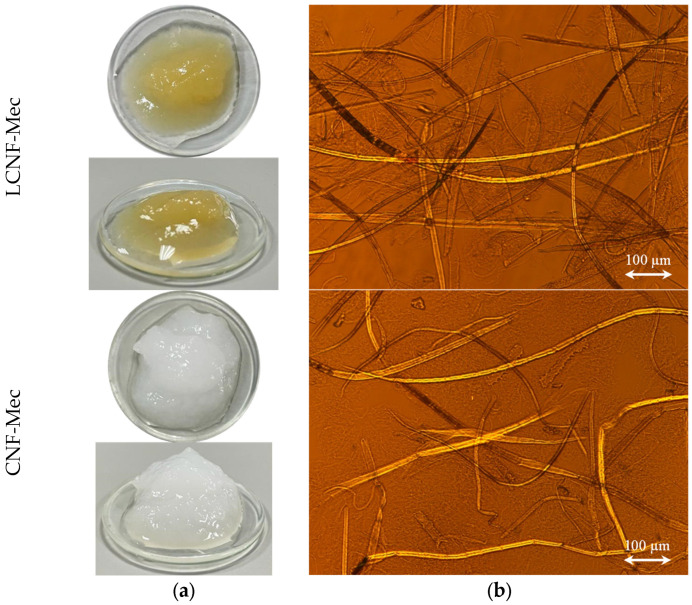
Visual observation (**a**) and optical microscope (**b**) images of (L)CNFs extracted from BS-UB and BS-B through chemical and mechanical pre-treatments.

The morphological features of NC isolated through different pre-treatments were assessed with visual analysis and an optical microscope (Figure 3). Visually, it is clearly shown (Figure 3a) that, apart from LCNF-Mec which exhibited a slight separation of phases, all NC formed a stable suspension in water with a gel-like state. Moreover, at 1%_DM_ solid content, the high transparency of the CNF-TO suspension is visibly observed, as expected from its fibrillation yield of about 90%. In general, considerable morphological differences of the NC occurred along with the type of pre-treatment applied [20]. Coherently with the nano-sized fraction and the nanofibrillation yield results, NC isolated with the mechanical pre-treatment showed a heterogeneous distribution with a long-shaped morphology (Figure 3b) with a length from 100 to 1000 μm and a width from 5 to 25 μm depending on the effect of the bleaching process. Moreover, NC isolated through the TEMPO-mediated oxidation pre-treatment had a shorter length, indicating that more severe damage occurred to the cellulose fibers [75]. Moreover, CNF-TO showed unique agglomerates of irregular particles with needle-like debris between them, suggesting significant size reduction due to excessive oxidation.

**Figure 4:**
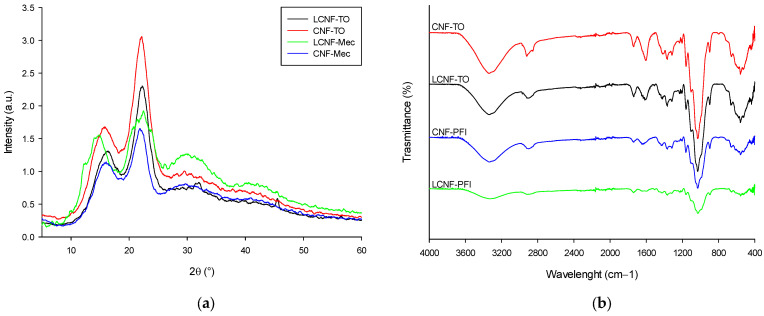
(L)CNFs’ XRD patterns (**a**) and FT-IR spectra (**b**).

XRD analysis (Figure 4a) revealed that all samples exhibited major peaks at 2θ = 16° and 22°, indicating the presence of a cellulose I_β_ structure [76], using Segal’s empirical method. Each pre-treatment affected the order of crystallinity and therefore the crystallinity index (CrI): LCNF-Mec < CNF-Mec < LCNF-TO < CNF-TO, with 48.83 ± 2.07%, 54.26 ± 1.87%, 58.30 ± 2.41%, and 61.97 ± 0.96%, respectively. It was noticeable that CNF-TO, which exhibited higher quality, had a higher crystallinity compared to the other NC. This increase in crystallinity can be attributed to the removal of impurities, such as amorphous non-cellulosic compounds (lignin and ashes), through a bleaching treatment and the decay of the amorphous region with a consequent rearrangement of the crystalline regions into a more ordered structure resulting from the oxidation reaction. The FT-IR spectra (Figure 4b) showed some signature characteristic bands of cellulose, including 3300, 2900, 1030, and 900 cm^−1^, which belonged to O–H stretching, C–H stretching, C–H deformation, C–O–C pyranose ring stretching vibration, and β-glycosidic linkages, respectively. All the spectra had a peak at around 1620 cm^−1^ attributed to the H–O–H bending of adsorbed water within the cellulose samples [77]. The highest peak intensity of both LCNF-TO and CNF-TO at around 1030 cm^−1^ could be attributed to the carbonyl bonds present in the cellulose skeleton [40].

### 2.3. (L)CNF-Based Aerogels Characterization

**Figure 5:**
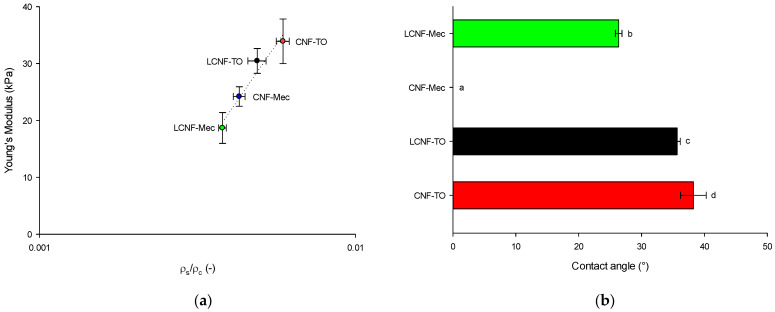
Young’s Modulus as a function of relative density (ρ_s_/ρ_c_) for (L)CNF-based aerogels (**a**). Dotted line represents the power law fitting curve (Equation (1)); average water contact angle of aerogels (**b**). Different letters denote significant differences (*p* < 0.05).

To establish a correlation between the QI of (L)CNFs and their potential applications, NC-based aerogels were synthesized to be applied in wastewater decontamination systems. To investigate the crucial factors for the handling and performance of adsorbent aerogels, the (L)CNF-based aerogels were obtained by freeze-drying hydrogels to preserve the internal pore structure. The characterization of these biomaterials encompassed assessments of the apparent density, the porosity, and the mechanical properties through compression tests (Table 4). The (L)CNFs exhibited excellent mechanical performance under an external pressure due to the high strength of NC and its stable porous structure, which is better than the traditional brittle SiO_2_ aerogels [31]. Moreover, the Young’s modulus of the (L)CNF-TO samples increased in respect to the (L)CNF-Mec ones, owing to the strong hydrogen bonding of the cellulose chains, which can facilitate the formation of a strong network. As a result, LCNF-TO and CNF-TO demonstrate a significant difference (*p* < 0.05) in compressive strength (of about ≈3 kPa) with respect to LCNF-Mec and CNF-Mec. Moreover, the tensile strength and compressive modulus have a direct relationship with the density of CNF-based aerogels [78]. The NC-based aerogels have the advantages to display a low density ranging from 5 to 9 kg/m^3^, and a corresponding porosity higher than 99%. The compressive behavior of aerogels, plotted against relative density in Figure 5a, is commonly described by the power law dependence of Equation (1) [79] as follows:

(1) $$E\propto {\left(\frac{\rho_{s}}{\rho_{c}}\right)}^{n},$$

where E is the Young’s Modulus, ρ_s_/ρ_c_ is the relative density, and n is the scaling exponent.

The power law predicts a scaling exponent of n = 3.708, suggesting that the obtained materials have an aerogel-like structure, which aligns well with the previous studies on cellulose [80,81]. To further validate their efficacy in dye removal from water, the aerogels were also characterized in terms of wetting properties (Figure 5b). Owing to the large number of hydrophilic hydroxyl groups in cellulose, the water contact angle for freeze-dried NC-based aerogels was lower than 40°, apart for CNF-Mec, into which a water droplet penetrates within 1 s, resulting in a water contact angle of 0°. Hence, water droplets cannot stand on the surface and are absorbed within 10 s, highlighting the hydrophilic properties of CNF-based aerogels.

### 2.4. Adsorption Behavior of (L)CNF-Based Aerogels

**Figure 6:**
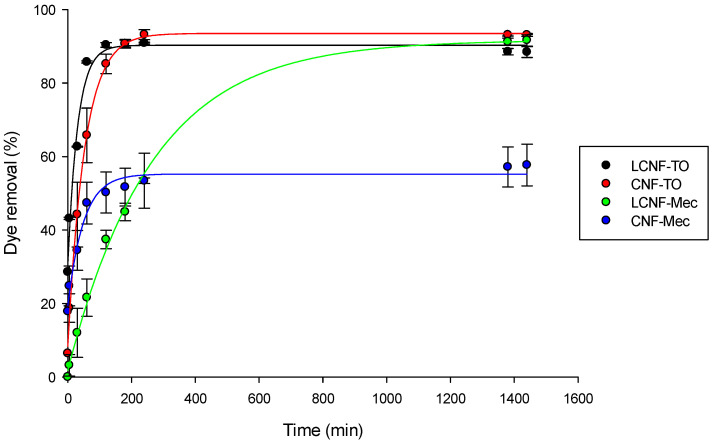
Effect of contact time on the adsorption capacity of MB cationic dye. Circles represent experimental data points and solid lines represent exponential decay fitting curves (Equation (2)).

The prepared adsorbent aerogels exhibited remarkable adsorption capabilities for methylene blue (MB) dye, attributable to their unique surface chemistry. Notably, (i) (L)CNFs possess abundant functional groups that engage with the cationic dye and (ii) the 3D porous structure of aerogels provides a large surface area, facilitating enhanced analyte–receptor interactions. Figure 6 illustrates the adsorption kinetics of different (L)CNF-based aerogels for a 10 mg/L MB solution. Initially, the results revealed that the adsorption rate increased rapidly due to the high availability of hydroxyl and carboxyl groups present in the aerogel (negative charge), which interact with the positively charged MB molecules. Subsequently, the adsorption rate gradually decreased as the adsorption sites within the adsorbent became saturated. Therefore, the affinity for adsorption by the (L)CNF-based aerogels was due to the hydroxyl and carboxyl functional groups [82], as highlighted by the FT-IR analysis (Figure 4). The adsorption equilibrium was reached at approximately 1, 4, 23, and 3.5 h after adsorption for LCNF-TO, CNF-TO, LCNF-Mec, and CNF-Mec, respectively. The slower adsorption observed with the CNF-Mec aerogel can be attributed to the lower surface charge resulting from the mechanical pre-treatment, as well as the lower QI value. Additionally, although LCNF-Mec and CNF-Mec have slightly different QI values, the presence of lignin content in LCNFs significantly influenced the adsorption properties through the formation of electrostatic interactions with the cationic dye [40].

Furthermore, all samples exhibited the exponential decay relationship (Equation (2)) between the adsorption time and percentage of dye removed. Therefore, Table 5 presents the relevant parameters of the selected kinetic model, which has a high R^2^ value; therefore, it is suitable for describing the adsorption behavior of the MB cationic dye.

(2) $$f\left(x\right)=y_{0}+a\cdot e^{-b\cdot x},$$

**Figure 7:**
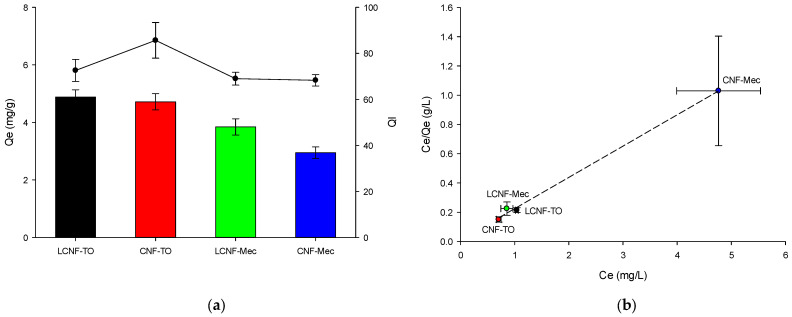
Effects of different aerogels on the MB adsorption capacity (left y-axis, Q_e_) and on the quality index (right y-axis, QI) (**a**). Langmuir isothermal adsorption model (**b**).

The high initial concentration of MB (10 mg/L) enhanced the adsorption capacity of the cellulose aerogels for the dye, as it increased the driving forces between the adsorbents and dye molecules. The LCNF-TO and CNF-TO-based aerogels allowed MB to reach the maximum equilibrium adsorption capacity (Figure 7a), because the (L)CNF-TO has higher specific surface area, leading to a higher affinity and interaction of carboxyl groups with MB. This result is also confirmed from visual observations (as shown in the graphical abstract), where it is clear visible how the MB solution became clearer after the treatment with aerogels. In addition, this finding indicated that (L)CNFs’ QI higher than 70% causes the adsorption process’s efficiency of MB to increase, reaching the maximum equilibrium adsorption capacity. Furthermore, the interaction between the adsorbent and adsorbate has been analyzed using the Langmuir adsorption isotherm model (Equation (12)) at 25 °C, as depicted in Figure 7b. The results demonstrated that NC-based aerogels have homogeneous adsorption characteristics for MB, with a maximum adsorption capacity of 4.68 mg/g. Moreover, the R_L_ value of MB adsorbed by (L)CNF-based aerogels was found to be less than 1 (Table 6), indicating a favorable adsorption process owing the strong intermolecular interaction between oxygen-containing groups of aerogels and hydrophilic segments of dye molecules.

## 3. Conclusions

NC-based aerogels with low density and high porosity represent a remarkable convergence of environmental remediation capabilities. In summary, NCs have been isolated from barley straw AFRs through chemical or mechanical pre-treatments and freeze drying to obtain a porous 3D network of aerogels. The observed relationship between the quality grade of various (ligno)cellulosic nanofibers and their adsorption behaviors underscore the significance of tailoring properties for enhanced performance as decontaminant biomaterials. The outcomes of the batch adsorption experiment underscore the high efficiency of (L)CNF-based aerogels in adsorbing cationic dye pollutants, such as MB dye, which could be ascribed to the internal porous structure and the electrostatic interaction between NC and cationic dye molecules. Notably, the achieved MB removal capacities consistently exceeded 90% for all samples, barring those derived from mechanically pre-treated NC with lower quality indices. Furthermore, the equilibrium data were consistent with the Langmuir isothermal model, and the maximum theoretical adsorption capacity for MB was determined to be 4.88 mg/g. In this scenario, NC-based aerogels with a QI higher than 70% hold great promise as effective adsorbent materials for wastewater treatment, owing to their low-cost and high adsorption capacity. Therefore, the integration of NC-based aerogels as innovative biomaterials marks a step forward in addressing water pollution and dye contamination.

## 4. Materials and Methods

### 4.1. Materials

Barley straw (BS), kindly provided by a farmer in Córdoba (Spain), was air-dried at room temperature and stored until usage. The relative humidity was 8.95 ± 0.23%.

The reactants used throughout this study were sodium hydroxide (NaOH, ≥99%, Sigma Aldrich, St. Louis, MO, USA), hydrochloric acid (HCl, 37%, Sigma Aldrich), sodium chlorite (NaClO_2_, ≥99%, Sigma Aldrich), sodium hypochlorite (NaClO, 10% *w*/*v* technical grade, PanReac, Barcelona, Spain), acetic acid (CH_3_COOH, ACS reagent, Sigma Aldrich), sodium bromide (NaBr, Hoynewell, Muskegon, NC, USA), TEMPO, 2,2,6,6-tetramethyl-piperidin-1-oxyle (C_9_H_18_NO, 98%, Sigma Aldrich), ethanol (C_2_H_5_OH, Sigma Aldrich), sulphuric acid (H_2_SO4, 95–98%, labbox, Barcelona, Spain), polymer polydiallyldimethylammonium chloride (BTG Instruments, Säffle, Sweden), Pes-Na (BTG Instruments, Säffle, Sweden), Copper(II) Ethylenediamine reagent (PanReac), and methylene blue (labbox).

### 4.2. Cellulose Fibers’ Isolation and Characterization

**Figure 8:**
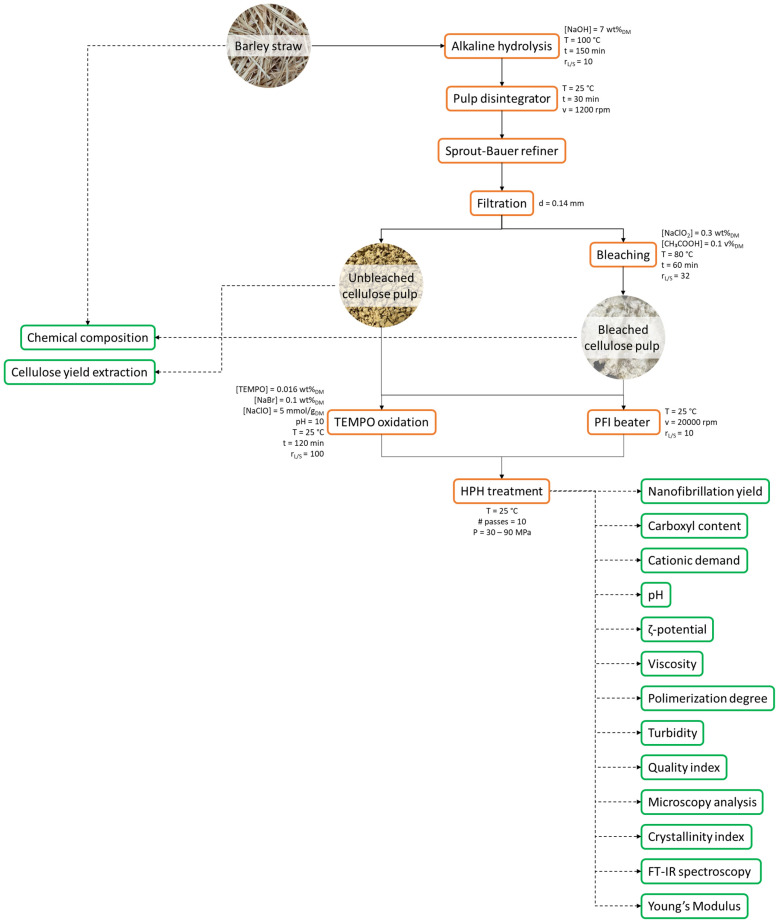
Flow chart of the cellulose and nanocellulose isolation and characterization.

BS was subjected to soda pulping process (7 wt% (on dry basis, DM) NaOH for 150 min at 100 °C with a liquid/solid ratio of 10:1) in a batch reactor equipped with an external vessel to maintain the desired temperature and a motor to ensure the rotation [40,60,83] to isolate unbleached cellulose pulp (BS-UB). Bleached cellulose fibers (BS-B) were obtained by repeating the bleaching process three times (0.3 wt%_DM_ NaClO_2_ in acidified conditions and 3 wt%_DM_ cellulose suspension for 1 h at 80 °C). Figure 8 schematically shows the flowchart of the cellulose fibers’ isolation and the characterization of obtained materials.

Cumulative yield of the pulping process and bleached treatment (%) was determined gravimetrically based on the isolated BS-UB and BS-B, respectively, relative to the initial dry mass of BS.

The chemical characterization of the BS, BS-UB, and BS-B was performed to determine the content of extractives (Tappi T-204), ashes (Tappi T-211), lignin (Tappi T-203os61), holocellulose (Tappi T-222), and α-cellulose (Tappi T-9m54) [84].

Furthermore, Fourier transform infrared spectroscopy (FT-IR) was carried out (FT-IR Spectrum Two series spectrophotometer, PerkinElmer, Waltham, Massachusetts, United States) to identify the functional groups and chemical structure at room temperature. The spectra were acquired in transmittance, with 40 scans collected over the wavenumber regions of 4000–400 cm^−1^ with a resolution of 4 cm^−1^.

The intrinsic viscosity (ɳ) of the cellulose fibers was determined according to the ISO 5351:2010 standard and converted in the degree of polymerization by Equations (3) and (4) [85] as follows:

(3) $$\mathrm{DP}\left(<950\right):\mathrm{DP}=\frac{{ɳ}_{s}}{0.42},$$

(4) $$\mathrm{DP}\left(>950\right):\mathrm{DP}=\frac{{ɳ}_{s}}{2.28},$$

The measurements were conducted five times, and the mean value and standard deviation were calculated.

### 4.3. Cellulose Nanofibers’ Isolation

BS-UB and BS-B were then subjected to nanofibrillation to obtain (ligno)cellulose nanofibers ((L)CNFs), respectively, using two pre-treatments methods according to the ISO 5264-2:2002 [86]: (i) chemical by TEMPO-mediated oxidation [71] and (ii) mechanical beating by PFI beater refining. Finally, 1 wt%_DM_ pre-treated fiber suspension was subjected to HPH treatment (PandaPlus 2000, GEA Niro, Düsseldorf, Germany) following 4 passes at 300 bars, 3 passes at 600 bars, and 3 passes at 900 bars [60]. The resulting (L)CNFs were designated as LCNF-TO, CNF-TO, LCNF-Mec, and CNF-Mec, depending on the starting cellulose pulp and pre-treatment method used.

### 4.4. Quality Index Determination and Cellulose Nanofibers’ Characterization

The comparison between the different (L)CNFs was made based on their quality index (QI), as proposed by Desmaisons et al. (2017) [28], which considers four characterization parameters. Once they are obtained, the calculation of the QI is made according to Equation (5) as follows:

(5) $$\mathrm{QI}=0.30\cdot x_{1}-0.03\cdot x_{2}-0.071\cdot x_{3}^{2}+2.54\cdot x_{3}-5.35\cdot \ln\left(x_{4}\right)+59.9,$$

where x_1_ is the nano-size fraction (%), x_2_ is the turbidity (NTU), x_3_ is the Young’s Modulus (GPa), and x_4_ is the macro-size (μm^2^).

The nanofibrillation yield and nano-sized fraction of (L)CNF suspensions on dry matter consists of the determination of the nanoscale particle fraction in the suspension gravimetrically, according to the protocol described by Besbes et al. [71] and Naderi et al. [87].

The turbidity of the (L)CNF suspensions was previously diluted to 0.1 wt%_DM_ and stirred for 10 min with T-25 Ultra Turrax device (IKA^®^ -Werke GmbH & Co. KG, Staufen, Germany) equipped with an S18N-19 G rotor and was measured with a portable turbidimeter (TN/3025 model, LabProcess, Barcelona, Spain). Three measurements were performed for each suspension and the results were expressed as the average with the sum of NTU (nephelometric turbidity units).

Young’s modulus of (L)CNFs was calculated from the stress–strain curves resulting from tensile tests performed on rectangular nanopapers samples (10 cm length and 15 mm width) using Lloyd LF Plus Tensile Test Machine (Lloyd Instruments Ltd., Bognor Regis, UK) equipped with a 1 kN load cell. The tests were conducted with initial gauge length of 10 cm and crosshead speed of 10 mm/min, following the standard NF Q03-004. All measurements were carried out at room temperature, and the results are presented as the mean value ± standard deviation based on nine repetitions. The nanopapers were prepared using the following procedure. (L)CNFs’ suspension was first dispersed in a pulp disintegrator to achieve a final concentration of 0.5 wt%_DM_. The suspension was then subjected to vacuum filtration at –600 mbar using a sheet former (Rapid Kothen, ISO 5269-2). Subsequently, the resulting sheet was dried at 85 °C between two nylon sieves and two cardboards (to prevent adherence) until nanopapers were completely dried. All nanopapers were then stored for 48 h in a conditioned room at 24 °C and 50% RH until future characterizations.

Optical images were collected using an optical microscope (Nikon Eclipse TE 2000S, Nikon instruments Europe B.V., Amsterdam, The Netherlands) coupled to a DS Camera Control Unit (DS-5M-L1, Nikon Instruments Europe B.V, Amsterdam, The Netherlands) to acquire and analyze images.

In addition to the QI, some other important parameters during (L)CNFs’ production were determined. The carboxyl content was determined using conductimetric titration [71,88]. The pH of (L)CNFs’ suspensions at 0.25 wt%_DM_ was adjusted at 2.7 using HCl solution (0.1 M) to replace the sodium cations bound to the carboxyl groups by hydrogen ions. The obtained suspension was titrated with 0.1 M NaOH. The presence of a strong and a weak acid in the titration curves corresponds to the excess of HCl and the carboxylate content, respectively. The average amount of –COOH groups was calculated using Equation (6) as follows:

(6) $$C_{\mathrm{COOH}}=\frac{\left(V_{2}-V_{1}\right)\cdot C_{\mathrm{NaOH}}}{g_{\mathrm{CNFs}}},$$

where C_COOH_ is the mmol of carboxyl content; V_2_ and V_1_ are the equivalent volumes of added NaOH solution; C_NaOH_ is the concentration of NaOH solution; g_CNFs_ is the weight of CNFs on dry basis.

The cationic demand was determined using a particle charge detector Mütek PCD 05 (BTG Instruments, Säffle, Sweden) following the methodology described by Espinosa et al. and Carrasco et al. [60,89]. Briefly, 15 mL of CNFs’ suspension at 0.2 wt%_DM_ was mixed with 25 mL of cationic polymer polydiallyldimethylammonium chloride (polyDADMAC 0.001 N) for 5 min with magnetic stirring. The supernatant recovered after centrifugation for 90 min at 4000 rpm was putted in the Mütek equipment, and anionic polymer (Pes-Na) was then added to the sample dropwise until the equipment reached the value of 0 mV. The volume of anionic polymer consumed was used to calculate the cationic demand using Equation (7) as follows:

(7) $$\mathrm{CD}=\frac{\left(C_{\mathrm{Ply}-\mathrm{DADMAC}}-V_{\mathrm{Poly}-\mathrm{DADMAC}}\right)-\left(C_{\mathrm{Pes}-\mathrm{Na}}-V_{\mathrm{Pes}-\mathrm{Na}}\right)}{g_{\mathrm{CNFs}}},$$

where CD is the cationic demand; V_i_ is the volumes of polymer used; C_i_ is the concentration of polymer; g_CNFs_ is the weight of CNFs on dry basis.

The ζ-potential was measured by dynamic light scattering (DLS) and electrophoretic mobility using a Zetasizer (ZSP, Malvern Instruments Ltd., Worcestershire, UK) at 25 °C. The (L)CNFs’ suspensions previously diluted to 0.1 wt%_DM_ with deionized water were stirred for 30 s with T-25 Ultra Turrax device (IKA^®^ -Werke GmbH & Co. KG, Staufen, Germany) equipped with an S18N-19 G rotor. The analysis was realized in triplicate and the average value with standard deviation was calculated.

The effect of the chemical and mechanical treatments used on chemistry structure of (L)CNFs was examined by FT-IR spectra on (L)CNFs previously dried in an air oven at 60 °C for 24 h, following the previously described methodology.

X-ray spectra of (L)CNFs were acquired using a Bruker D8 Discover equipped with a monochromatic Cu Kα1 source over an angular range of 7–50° at a scan speed of 1.56°/min. The crystallinity index (CI) was calculated by using Equation (8) [90] as follows:

(8) $$\mathrm{CI}=\frac{I_{200}-I_{\mathrm{am}}}{I_{200}}\cdot 100,$$

where I_200_ is the intensity of the 200 peak (I_200_ 2θ = 22°) and Iam is the intensity minimum between the peaks at 200 and 110 (I_am_ 2θ = 15°).

The intrinsic viscosity of 0.2 wt%_DM_ of (L)CNFs’ suspensions was determined according to the ISO 5351:2010 standard and the degree of polymerization is related to the intrinsic viscosity (as reported in the previous section).

### 4.5. (L)CNF-Based Aerogels Preparation and Characterization

(L)CNFs’ suspensions at 0.5 wt%_DM_ were frozen for 24 h and then freeze dried at –85 °C under 0.5 mBar for 72 h in a Lyoquest -85 lyophilizer (Telstar, Terrassa, Spain).

The porosity, a key property in the formation of aerogels due to its relationship with adsorption capacity [44], is calculated using the following equation [40]:

(9) $$P=\left(1-\frac{\rho_{s}}{\rho_{C}}\right)\cdot 100,$$

where ρ_S_ is the apparent density of aerogels and ρ_C_ is the density of cellulose (considered as 1540 kg/m^3^).

The mechanical properties of aerogels (cylinder with 28 mm diameter and 24 mm heigh) were evaluated using Lloyd LF Plus Tensile Test Machine (Lloyd Instruments Ltd., Bognor Regis, UK) equipped with a load cell of 1 kN by compression tests with a strain limit of 80% at a speed of 2 mm/min.

The water contact angle (WCA) was measured in a contact angle goniometer (Ossila Ltd., Sheffield, UK) using the sessile drop method. A 10 µL droplet of deionized water was casted on the surface of the aerogel and the angle was measured from 0 to 10 s. The measurements were replicated 5 times, and the results were expressed as averages ± standard deviation.

### 4.6. Dye Removal Efficiency of (L)CNF-Based Aerogels

Methylene blue (MB) was employed as typical cationic dye to simulate wastewater. All adsorption tests were performed by immersing the aerogels in 50 mL of 10 mg/L MB aqueous solution. During 24 h of adsorption, the change of dye concentration in the solution was analyzed by UV-vis spectrophotometer (Lambda 25, Perkin Elmer Inc, Waltham, MA, USA) using a calibration curve obtained from the linear fitting (A_664 nm_ = 0.2075 · C_MB_, R^2^ = 0.9991) of the measured absorbance (A_664 nm_) as a function of MB concentration (ranging from 0 to 10 mg/L). The percentage of dye removal during the adsorption kinetic was calculated using the following relationship:

(10) $$\%\;\mathrm{Dye}\;\mathrm{removal}=\left(\frac{C_{i}-C_{t}}{C_{i}}\right)\cdot 100$$

where C_i_ and C_t_ are the initial and after time t concentrations of dye (mg/L), respectively.

The amount of dye adsorbed Q_e_ (mg/g) onto the aerogel was calculated from the mass balance equation as follows:

(11) $$Q_{e}=\frac{C_{i}-C_{e}}{M}\cdot V$$

where C_e_ is the equilibrium concentration of dye (mg/L), V is the volume of dye solution (L), and M is the mass of the adsorbent aerogel used (g).

Langmuir isotherm (Equation (12)) is used to describe adsorption equilibrium type and maximum adsorption capacity [57].

(12) $$\frac{C_{e}}{Q_{e}}=\frac{1}{Q_{m}}\cdot C_{e}+\frac{1}{Q_{m}K_{L}},$$

where Q_m_ is the maximum adsorption capacity (mg/g) and K_L_ is the Langmuir constant (L/mg).

Furthermore, the separation factor (R_L_), calculated as in Equation (13), is used to describe the essential characteristic of Langmuir isotherm: irreversible (R_L_ = 0); favorable (0 < R_L_ < 1); linear (R_L_ = 1); unfavorable (R_L_ > 1) [91].

(13) $$R_{L}=\frac{1}{1+K_{L}\cdot C_{i}},$$

### 4.7. Statistical Analysis

The experiments were conducted in triplicate, and the results were presented as means ± standard deviation. Significant difference of *p* < 0.05 was taken in SPSS 20 statistical software (SPSS Inc., Chicago, IL, USA) through a one-way analysis of variance (ANOVA) followed by Tukey’s test.

## Figures and Tables

**Table 1:** Chemical characterization of barley straw (BS), barley straw unbleached pulp (BS-UB), barley straw bleached pulp (BS-B), and cellulose extraction yield.

		BS	BS-UB	BS-B
Extractives in water	(%)	14.60 ± 0.50 ^c^	4.40 ± 0.05 ^b^	3.53 ± 0.3 ^a^
Extractives in EtOH	(%)	10.24 ± 0.24 ^a^	13.91 ± 0.11 ^c^	12.83 ± 0.50 ^b^
Ashes	(%)	7.38 ± 0.04 ^c^	1.41 ± 0.01 ^b^	0.82 ± 0.01 ^a^
Lignin	(%)	11.88 ± 1.45 ^b^	10.30 ± 0.70 ^b^	1.09 ± 0.04 ^a^
Hemicellulose	(%)	22.80 ± 0.59 ^b^	21.48 ± 0.13 ^a^	21.73 ± 0.16 ^a^
α-cellulose	(%)	34.86 ± 0.33 ^a^	47.41 ± 0.70 ^b^	60.66 ± 0.48 ^c^
Cellulose extraction yield	(%)	–	33.41 ± 3.67 ^b^	26.85 ± 2.75 ^a^

Different letters denote significant differences (*p* < 0.05) among the different samples within each row (n = 3).

**Table 2:** Chemical characterization of (L)CNFs particles.

		LCNF-TO	CNF-TO	LCNF-Mec	CNF-Mec
Nanofibrillation yield	(%)	61.24 ± 1.00 ^c^	89.70 ± 0.87 ^d^	16.08 ± 0.35 ^a^	26.17 ± 0.45 ^b^
Cationic demand	(µeq/g)	624.80 ± 35.15 ^b^	732.40 ± 40.05 ^c^	292.96 ± 21.82 ^a^	333.49 ± 37.29 ^c^
Carboxyl content	(µmol/g)	431.11 ± 109.03 ^c^	653.06 ± 41.72 ^b^	153.32 ± 11.33 ^a^	157.55 ± 88.60 ^a^
ζ-potential	(mV)	–19.13 ± 6.92 ^b^	–63.97 ± 4.41 ^a^	–20.00 ± 0.75 ^b^	–25.77 ± 1.21 ^b^
pH	(-)	7.56 ± 0.22 ^b^	7.16 ± 0.15 ^b^	6.48 ± 0.16 ^a^	6.27 ± 0.35 ^c^
Viscosity	(mL/g)	225.15 ± 23.20 ^a^	189.69 ± 28.71 ^a^	518.09 ± 14.36 ^b^	512.78 ± 23.21 ^b^
Polymerization degree	(-)	536.07 ± 55.23 ^a^	451.65 ± 68.36 ^a^	1233.56 ± 34.18 ^b^	1220.90 ± 55.26 ^b^
Turbidity	(NTU)	56.95 ± 2.47 ^b^	15.02 ± 3.44 ^a^	289.50 ± 9.19 ^d^	189.05 ± 10.39 ^c^
Young’s Modulus	(MPa)	30.08 ± 0.26 ^c^	37.27 ± 1.24 ^d^	9.59 ± 1.04 ^b^	4.86 ± 0.68 ^a^

Different letters denote significant differences (*p* < 0.05) among the different samples within each row (n = 3).

**Table 3:** Fitting parameters and coefficient of determination for the fitting of the data in Figure 2, using the linear equation f(x) = y_0_ + a∙x.

Parameters	Carboxyl Content	Cationic Demand
y_0_	6.038	190.023
a	7.096	6.333
R^2^	0.986	0.976
Adj R^2^	0.979	0.964

**Table 4:** Mechanical properties of (L)CNF-based aerogels.

		LCNF-TO	CNF-TO	LCNF-Mec	CNF-Mec
Young’s Modulus	(kPa)	30.46 ± 2.19 ^c^	33.91 ± 3.92 ^c^	18.68 ± 2.71 ^a^	24.21 ± 1.72 ^b^
Tensile strength	(kPa)	2.78 ± 0.32 ^b^	3.09 ± 0.17 ^b^	1.81 ± 0.05 ^a^	1.61 ± 0.18 ^a^
Stiffness	(kN/m)	6.96 ± 0.48 ^b^	7.23 ± 0.16 ^b^	4.73 ± 0.85 ^a^	5.51 ± 0.78 ^a^
Apparent density	(kg/m^3^)	7.53 ± 0.50 ^c^	9.08 ± 0.43 ^d^	5.78 ± 0.40 ^a^	6.61 ± 0.16 ^b^
Porosity	(%)	99.51 ± 0.03 ^b^	99.41 ± 0.03 ^a^	99.62 ± 0.03 ^d^	99.57 ± 0.01 ^c^

Different letters denote significant differences (*p* < 0.05) among the different samples within each row (n = 3).

**Table 5:** Kinetic coefficients and fitting parameters for dyes adsorption model using Equation (2).

Parameters	LCNF-TO	CNF-TO	LCNF-Mec	CNF-Mec
y_0_	1.042 ± 0.025	0.708 ± 0.042	0.857 ± 0.111	4.765 ± 0.774
a	9.250 ± 0.484	9.283 ± 0.557	9.028 ± 0.661	5.231 ± 0.359
b	0.346 ± 0.004	0.043 ± 0.013	0.005 ± 0.001	0.377 ± 0.013
R^2^	0.999 ± 0.001	0.997 ± 0.001	0.994 ± 0.004	0.947 ± 0.006
Adj R^2^	0.998 ± 0.001	0.996 ± 0.001	0.992 ± 0.006	0.928 ± 0.008

**Table 6:** Langmuir isothermal adsorption fitting parameters of Equation (13).

Parameters	Value
Q_m_	4.677
K_L_	19.796
R^2^	0.997
Adj R^2^	0.996

## Data Availability

All data and materials are available on request from the corresponding author. The data are not publicly available due to ongoing research using a part of the data.
